# Enhanced stress-resilience training for surgical trainees

**DOI:** 10.1093/bjsopen/zrab054

**Published:** 2021-07-29

**Authors:** O W Luton, O P James, K Mellor, C Eley, L Hopkins, D B T Robinson, C C Lebares, A G M T Powell, W G Lewis, R J Egan

**Affiliations:** 1 Health Education and Improvement Wales’ School of Surgery, Tŷ Dysgu, Cefn Coed, Nantgarw, UK; 2 University of California, San Francisco, California, USA; 3 Division of Cancer and Genetics, Cardiff University, Heath Park, Cardiff, UK; 4 Department of Surgery, Morriston Hospital, Swansea Bay University Health Board, Swansea, UK; 5 Swansea University, Swansea, UK

## Abstract

**Introduction:**

Core surgical training programmes are associated with a high risk of burnout. This study aimed to assess the influence of a novel enhanced stress-resilience training (ESRT) course delivered at the start of core surgical training in a single UK statutory education body.

**Method:**

All newly appointed core surgical trainees (CSTs) were invited to participate in a 5-week ESRT course teaching mindfulness-based exercises to develop tools to deal with stress at work and burnout. The primary aim was to assess the feasibility of this course; secondary outcomes were to assess degree of burnout measured using Maslach Burnout Inventory (MBI) scoring.

**Results:**

Of 43 boot camp attendees, 38 trainees completed questionnaires, with 24 choosing to participate in ESRT (63.2 per cent; male 13, female 11, median age 28 years). Qualitative data reflected challenges delivering ESRT because of arduous and inflexible clinical on-call rotas, time pressures related to academic curriculum demands and the concurrent COVID-19 pandemic (10 of 24 drop-out). Despite these challenges, 22 (91.7 per cent) considered the course valuable and there was unanimous support for programme development. Of the 14 trainees who completed the ESRT course, nine (64.3 per cent) continued to use the techniques in daily clinical work. Burnout was identified in 23 trainees (60.5 per cent) with no evident difference in baseline MBI scores between participants (median 4 (range 0–11) *versus* 5 (1–11), *P* = 0.770). High stress states were significantly less likely, and mindfulness significantly higher in the intervention group (*P* < 0.010); MBI scores were comparable before and after ESRT in the intervention cohort (*P *=* *0.630, median 4 (range 0–11) *versus* 4 (1–10)).

**Discussion:**

Despite arduous emergency COVID rotas ESRT was feasible and, combined with protected time for trainees to engage, deserves further research to determine medium-term efficacy.

## Teaser

This study aimed to assess the influence of a novel Enhanced Stress and Resilience Training (ESRT) course delivered at the start of core surgical training in a single UK Statutory Education Body. Five weeks of ESRT programme with home practice was offered to surgical trainees and deemed feasible and acceptable. Results showed significantly reduced perceived stress and increased mindfulness at short-term follow-up in the intervention cohort. Further studies will be required to measure the efficacy of ESRT.

## Introduction

Stress at work is common and defined as a bodily or emotional event that causes physical or mental conflict. The key unanswered question is how to measure and counter it reliably^1^.

The current demands of front-line clinical work allied to the surgical training curriculum drive pressure and emotional stress, which not only affect the wellbeing of trainees, but probably also influence key clinical performance indicators, and ultimately threaten patient safety. The global COVID-19 pandemic has resulted in worldwide healthcare burdens, seldom, if ever, witnessed before. The stress of working in clinical areas overwhelmed with COVID-19, combined with reduced elective operating and fewer training opportunities, make the present times the most challenging in living memory for trainees. This risks workforce attrition at a time that healthcare systems can least afford^2–4^.

Prior to the current pandemic, burnout in trainees was increasingly recognized with manifestations similar to those seen in conditions such as acute stress reaction and post-traumatic stress disorder^5^. As an entity, burnout is associated with untold negative physical, mental health and social consequences, such as ischaemic heart disease, stroke, substance abuse, depression, suicide and divorce^6–8^. Burnout is reported among 60 per cent of surgical trainees in Wales, with core surgical trainees (CSTs) at most risk; these figures are in keeping with those reported in US general surgery residents and emergency physicians^9^^,^^10^.

Among US surgical trainees, high dispositional mindfulness decreases these risk factors by 75 per cent or more^11^, and formal mindfulness training has been shown to be feasible and acceptable^12^^,^^13^. In other high-stress populations, such as the US Marine Corps, the police and elite athletes^14–17^, formal mindfulness training has improved wellbeing, stress, cognition and performance, yet only a modest number of studies have hypothesized the ability of such training to mitigate stress and burnout across medical specialties, or to effect improvements in the cognition^18^ and performance^19^ of physicians. To address these divergences and thereby promote the wider adoption of contemplative practices within medical training, investigators have developed enhanced stress-resilience training (ESRT), which is streamlined, tailored and contextualized for clinicians. ESRT is a modified form of mindfulness-based stress resilience, the most scientifically studied mindfulness training, developed by Jon Kabat-Zinn^20^ in the 1980s. The aims of the present study were to assess the feasibility of an ESRT curriculum programme delivered to a cohort of CSTs in a single UK statutory education body (SEB).

## Methods

A voluntary, non-randomized sample of newly appointed CSTs attending a core surgical boot camp^21^ in September 2020 were approached for inclusion in this study. Written informed consent was obtained prior to participation.

The participation cohort was divided into two groups alphabetically to facilitate assignment to ESRT tutors. Data were collected using pre- and postintervention questionnaires comprising multiple psychological assessment inventories at baseline (T1) and repeated at 8 weeks following completion of ESRT (T2).

Commencing with a 90-minute introduction session at the national core surgical training boot camp, ESRT was delivered over 5 weeks as 75-minute online tutorials incorporating 15 minutes of debrief prior to the assigned 60-minute ESRT.

Classes focused on developing mindfulness skills (sustained attention and open monitoring) in addition to training focused on stress and coping techniques. ESRT also involves a practice component: up to 20 minutes per day of mindfulness exercises following guided media and an emphasis on ‘informal’ practices in small recurrent ways throughout every day. Protected time for ESRT was unavailable due to service provision and training commitments so classes were delivered regularly out of work every Tuesday or Thursday between 18.30 and19.45 hours according to group assignment. The study protocol is available in the supporting information. All aspects of the intervention and assessment were approved by the Health Research Authority (IRAS ID 278852).

### Outcome measures

The primary hypothesis was that ESRT is feasible for CSTs and can form part of a prescribed curriculum. Feasibility was assessed using qualitative data collected from questionnaires (Likert scale, psychological assessment tools and free text), session attendance (course completion/drop-out ratio) and interviews (post-course completion discussion with trainees) by two researchers and assessed against six major feasibility domains: demand, implementation, practicality, adaptation, integration and acceptability. This method was chosen to allow comparison with previously reported studies^13^.

Demand was defined as the perceived need for ESRT within the cohort of CSTs by both trainees and trainers. This was evaluated by discussions with relevant surgical trainers^3^, ESRT instructors^2^, an international expert in ESRT implementation^1^ and the Head of School of Surgery^1^ responsible for curriculum delivery, to identify interest and potential barriers. Implementation was defined as the process required to embed ESRT seamlessly into already overcrowded curricula. Practicality was defined by overall cost of time and finances associated with the ESRT programme. This was evaluated using specialty trainee manager cost reports, instructor attendance forms and follow-up questionnaires. Adaptation was predefined prior to intervention administration. ESRT is a codified curriculum designed to address the unique challenges faced by surgical trainees. Integration was defined as the use of ESRT skills outside of the programme and continued use at follow-up. It was measured by questionnaire response in particular with regard to examples where ESRT was successfully used in clinical settings. Acceptability was defined as perceived appropriateness and satisfaction of programme delivery based on qualitative follow-up questionnaire data.

A secondary hypothesis to be further analysed in follow-up work was that cognitive skills developed by ESRT will result in beneficial effects apparent in multiple domains, assessed using validated questionnaires, including burnout (Maslach Burnout Inventory^22^ (aMBI)), stress (Perceived Stress Scale^23^ (PSS-10)), and psychosocial wellbeing (Patient Health Questionnaire-2^24^ (9PHQ-2), Cognitive and Affective Mindfulness Scale – Revised^25^ (CAMS-R)) and anxiety (State—Trait Anxiety Inventory^26^ (STAI-6)). These assessment tools have been validated against an age-matched population of US surgical residents^11–13^.

### Analysis

Statistical analysis appropriate for non-parametric data were performed using SPSS® version 26 (IBM Corp, Armonk, New York, USA). Mann–Whitney *U* tests were used to compare prevalidated psychological assessments at two separate time points (T1 and T2).

## Results

**Fig. 1:**
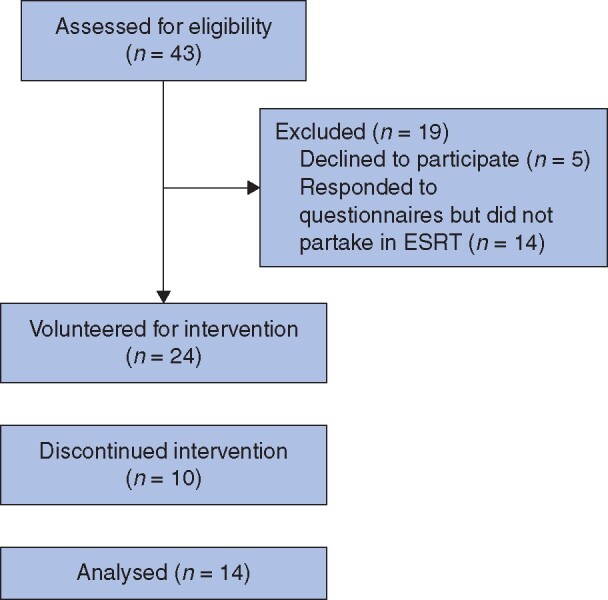
Enhanced stress-resilience training participation flow diagram

Of 43 CST boot camp attendees, 38 completed pre- and postintervention questionnaires, with 24 choosing to participate in the ESRT programme. Of the 24 who committed to ESRT, 10 did not complete ESRT satisfactorily, resulting in 14 CSTs remaining for analysis in the intervention group (58 per cent) (*Fig. 1*).

Demographics of participants in non-intervention, non-completion and intervention groups are shown in *Table 1*.

**Table 1:** Group demographics

Demographics	Intervention (*n* = 14)	Non-completion (*n* = 10)	Non-intervention (*n* = 14)
**Male**	9	4	10
**Female**	5	6	4
Age (years)*	28 (25–32)	29 (26–34)	29 (26–35)
**IMG**	3	4	5
**Tertiary centre**	6	5	9
**Subspecialty**			
General surgery	6	4	5
T&O	5	1	3
Other	3	5	6

*Values are median (range). IMG, international medical graduate; T&O, trauma and orthopaedic surgery.

### Primary outcome – feasibility

*Table 2* shows trainee comments assigned to each feasibility domain at T2.

**Table 2:** Trainee comments from T2 follow-up questionnaire

Feasibility domain	Trainee comments
**Demand**	‘Trial of novel techniques for stress management.’ ‘Explore whether mindfulness practice could reduce stress, benefit wellbeing.’ ‘I believe our training programme can be very stressful at times, both within clinical duties and outside of work trying to pass exams and improve your CV. I think that any tools to help deal with this stress are important.’
**Implementation**	‘Very well run with experienced therapists, sessions ran smoothly, the benefit of these sessions over zoom was limited – it made it hard to engage as a beginner to mindfulness.’ ‘Although there were difficulties with regards to the pandemic I think that the zoom sessions were a successful way to run the sessions. Personally I think I could relax more in the sessions being in my own environment.’ ‘I think that trying to implement a programme for a large number of individuals, all of whom work varying shifts was always going to be very challenging. I’m sure very few people were able to make all scheduled sessions due to other work commitments, but I do think the implementation was appropriate. I think the programme requires a level of trust with your other participants so I found keeping the same group for the entire programme beneficial.’
**Practicality**	‘Free course was great, easy to access, large time commitment, can benefit from only small amounts of practice a day when suited.’ ‘There was some difficulty in fitting in the sessions around the on-call rota but having two options per week made it manageable.’ ‘ESRT did not incur any monetary cost. I found that from a time value perspective, considering that this was an investment into my betterment in practice as well as my future, I did not perceive the time contributed to this programme as a cost or a loss of time.’
**Integration**	‘Mindful practice in theatre refocused the mind, prior to stressful situations on night shifts, difficult discussion with families or receiving criticism.’ ‘During on calls I will use grounding when walking between wards to keep a handle of rising stress. I also used it when sitting a recent exam to stop myself panicking.’ ‘During an on-call shift it felt like my bleep was going off every 2 minutes and my jobs list was getting longer and longer. I used some of the ESRT techniques very briefly to calm myself down and it allowed me to prioritize the tasks and get on with ticking them off.’
**Acceptability**	‘Personally, I thought it was appropriate to give us a tool set to handle stress as long as this is done in tandem with the programme organizers looking at ways to reduce modifiable stressors within the programme.’ ‘Grateful for the opportunity to try ESRT. Outcome – I exercise a lot, and I find that going out for a cycle/going on bike trainer for an hour in evening probably fulfils similar de-stressing/detachment time as is intended with ESRT.’ ‘I felt ESRT helped me in such way that I gradually learned my awareness of thoughts and reaction to them during a stressful condition are observable and modifiable. It is immensely helpful for mindfulness and enjoying the moments as we go through ups and downs during our career and life.’

#### Demand

Qualitative data revealed themes alluding to trainees wanting formalized methods to manage stress, to care for their mental health and engage in mindfulness.

#### Implementation

Barriers identified included isolation restrictions due to COVID-19, the lack of protected delivery time and an already arduous CST curriculum.

Course delivery was via an online distance-learning tool which eliminated geographical isolation but raised issues with technological deficiencies and depersonalization. Of the 10 trainees who dropped out of ESRT, nine cited the ESRT timing outside of working hours as the main cause.

Facilitators identified included both trainer leadership and trainee enthusiasm for ESRT, flexibility of the above to deliver ESRT remotely in challenging times and the feeling of trust building within groups.

No curriculum, examination or surgical training was affected by the delivery of the programme.

#### Practicality

Despite initial high attrition rates (42 per cent dropout within week one, 10 trainees), subsequent attendance rates were very high (absence rate 16.6 per cent (14 of 84 sessions) in the intervention group). Absences were attributable to service provision commitments in 86 per cent of cases (12 of 14). Time practised per day was very variable but ranged between 2 and 20 minutes for most trainees. Days practised per week were again variable, but most trainees made conscious efforts to set dedicated practice time (2 to 4 times weekly).

The outlay for the School of Surgery consisted of 70 hours relating to meetings and course development and €3500(ESRT instructors and materials). Each ESRT instructor committed approximately 22 hours to developing and delivering the curriculum, whilst CSTs completing the course committed 8 hours of formal teaching time (in addition to the boot camp), and 5 (2–13) hours to practise. No costs were incurred by CSTs.

Qualitative data reflected challenges administering this course and highlighted arduous rotas and paucity of time, because of the academic demands faced by CSTs, as a major barrier to practicality.

#### Adaptation

Necessary adaptations for study purposes were limited to programme delivery (as trainers were unable to attend the introduction session at the boot camp in person, subsequent sessions were delivered remotely), session delivery (sessions were run on week-night evenings due to the inability to provide protected teaching time in working hours), and session timing (allowing trainees the opportunity to debrief before the scheduled session).

#### Integration

Of the 14 volunteers in the intervention group, nine (64 per cent) continued to use the techniques in daily clinical work at 8-week follow-up (T2).

#### Acceptability

Despite the above challenges, of the original 24 CST volunteers, 22 (92 per cent) considered the course valuable and unanimous support was expressed to continue the programme in future.

Based on these domains, ESRT was judged feasible for CSTs.

### Secondary outcomes – psychological assessment

#### Burnout

Burnout was identified in 23 (61 per cent) of the trainees who completed the questionnaires. There was no evident difference in baseline aMBI scores between trainees who participated and those who did not (median 4 (range 0–11) *versus* 5 (1–11), *P* = 0.770). There was no statistically significant improvement in aMBI scores between the pre- and postintervention groups (4 (range 0–11) *versus* 4 (1–10), *P* = 0.630).

#### Stress

High (7 trainees, 18 per cent), moderate (27 trainees, 71 per cent) and low (4 trainees, 11 per cent) levels of stress were identified in all trainees who completed the questionnaires at T1, using PSS-10. When directly comparing levels of perceived stress at T2 between groups, levels of high stress were three-fold higher in the non-intervention group (6 *versus* 2) and low levels of stress were six-fold greater in the intervention group (6 *versus* 1). The median score was 13 (range 8–33) in the intervention group *versus* 26 (11–34) in the non-intervention group at T2 (*P* < 0.010).

#### Mindfulness

There was a significant increased level of mindfulness, measured with CAMS-R, within the intervention group from T1 to T2 (median score 18 (range 6–26)*versus* 22 (14–28), *P* = 0.010). There was no difference in mindfulness from T1 to T2 for the non-intervention group (median score 16 (range 9–22) *versus* 16 (8–23)).

#### Depression

A potential major depressive disorder was identified in six trainees at T1 (intervention group 1 trainee, non-intervention group 4 trainees, non-completion group 1 trainee). Of 38 trainees who completed the questionnaires, five expressed thoughts of suicidal ideation for several days of the month. There was no significant difference in potential depressive disorders seen between either group at T1 or T2. There was, however, a statistically significant positive correlation (*rho* 0.481, *P* = 0.002) between depression (PHQ-2) and burnout (aMBI) scores. Neither of these scores was affected by ESRT in the short-term feasibility follow-up period.

#### Anxiety

High levels of anxiety were identified in four (10.5 per cent) trainees at T1 using STAI-6. There was no difference in level of anxiety between groups at T2: intervention group (median score 5 (range 0–15)) *versus* non-intervention group (6 (2–15), *P* = 0.450).

## Discussion

The principal finding of this study was that ESRT is feasible and can be implemented across a wide geographical region. The initiative was welcomed by most of the target cohort, and the remote online format was well received. Moreover, participants integrated the skills learned and continued their use outside clinical work. In contrast, important challenges were identified with elements of delivery. Attrition during the first week was high (10 trainees, 42 per cent) and was attributed to inconvenient timing and perceived clashes with rostered duties. Future adaptations should consider these factors, with a strong case for allocation of protected time, although this also has implications regarding sustainability.

Notwithstanding the concept of managing burnout, mindfulness training among surgeons in the UK has received only casual interest^27^, arguably because of a misunderstanding regarding the potential prolonged benefits, which is often perceived to be a form of real-time relaxation rather than a life-learned skill to develop resilience. This study draws comparisons with the reports by Lebares and colleagues related to a pilot RCT involving 21 US surgical residents^12^^,^^13^. In keeping with the findings of the present study, ESRT was found to be not only feasible, but also associated with less perceived postintervention levels of stress (η^2^ = 0.07) and more mindfulness (η^2^ = 0.13). Similar to the present study, ESRT had little effect on burnout.

In practical terms, the decision to provide ESRT via an online platform was mandated by the COVID-19 worldwide pandemic, enforcing social distancing regulations. When reviewing other forms of resilience training provided online or via an ‘app’, results appear less effective than those observed after in-person training^28^, which may influence psychological inventory scores. Provision of a bespoke ESRT course carries financial and manpower implications (time) and, although much of this initial outlay should be an isolated investment, this element requires careful consideration by educational bodies wishing to provide this innovation. Although beyond the scope of this study, on the balance of evidence such investment should deliver handsome returns with less potential absenteeism and training programme attrition.

Sixty per cent of the newly appointed CSTs reported burnout in this cohort which is in keeping with historic cohorts from the Wales SEB. On multivariable analysis, burnout, in all three domains assessed, was associated with the CST cohort more than with any other trainee grade^10^. This is worrisome, because at this stage of training (after completion of the foundation programme, akin to internship in North America), success in attaining a training number for a CST programme has been reported to be associated with widespread prevalence of burnout. Of more concern was the finding that five trainees (13 per cent) entering the training programme reported and displayed features of depression and suicidal tendencies. Moreover, significant positive correlations were observed between symptoms of depression (PHQ-2 score) and burnout (aMBI score).

A reciprocal relationship between burnout, depression and high or overwhelming stress has been reported previously, although causality is unclear^12^. The unexpected observation of significant improvements in intervention stress scores, in a pilot study with short follow-up, suggests that effects on burnout and depression should be better explored in an adequately powered prospective study.

This study has several inherent limitations. It was set up as a feasibility study, encompassing a modest sample size, consisting of volunteers from a single SEB, with no randomized protocol. The intention to treat was to include all participants in the intervention and, consequently, caution needs to be exercised in any subsequent comparison between participants and non-participants. It is possible that the reasons non-participants were not able to take part may be the same reasons why their results at the designated measurement points differed. Analysis of qualitative data has been carried out using a deductive method; the potential drawback to this is that it does not allow for new themes to emerge.

The effects of the COVID-19 pandemic are likely to have influenced the metrics reported by trainees, who worked emergency COVID rosters, witnessed vanishing training opportunities, and were redeployed to the front line in intensive care, internal medicine and emergency departments. COVID-19 also resulted in adaptation and restricted access to resources, which would otherwise be available. Some of these limitations are offset by the novelty of the study, its clinical importance with regards to trainee health and wellbeing, and its role in highlighting important working environment upgrades that are clearly required within medicine and surgery.

Demand for an intervention to address the effects of burnout among clinicians is self-evident and unequivocal, but delivering such innovative programmes has challenges. To date, translational leadership has driven change, but this is not enough. For institutional change to occur, financial resource is required, in addition to protected teaching time, and most importantly engagement by both trainees and trainers alike. An appropriately powered RCT will be required to assess the efficacy of ESRT formally within surgery. From a healthcare perspective, 2020 was a year like no other, although it may yet be surpassed by 2021 in terms of managing a high-stress, craft specialty workforce, within the constraints of a system driven to the brink of collapse by the worst health crisis in living memory. Never has there been a more apt time to push the agenda of protecting the next generation of medical professionals from the potential harm that modern training systems convey.

## Collaborators

M. Abukhder; R. Allen; O. Allon; A. Beveridge; A. Burgan; S. Canbilen; S. Choi; H. Collins; P. Cripps; E. Daketsey; I. De Gruttola; J. Faiz; C. Foo; E. Hadjikyriacou; K. Harborough; J. Hayes; C. Holden; S. Latif; H. Li; A. Lukaszewicz; I. Mahamud; N. Mallya; R. Mann; S. Maryosh; G. Michael; R. Mistry; M. Yaseen; R. Morris; A. Naisbitt; K. Oo; S. Rajendran; E. Sams; N. Shivakumar; A. Siddiqui; W. Thompson; D. Warrell; L. Williams; A Zander.

## Supplementary Material

zrab054_Supplementary_Data
