# Supplementary material for: Enhanced stress-resilience training for surgical trainees
Source: BJS Open. 2021 Jul 29;5(4):zrab054. doi: 10.1093/bjsopen/zrab054 (PMC8320339; doi:10.1093/bjsopen/zrab054)
Supplement: zrab054_Supplementary_Data [file zrab054_supplementary_data.pdf]

Enhanced Resilience Training to Improve Mental Health, Stress, and Performance in Core Surgical Trainees

Investigators: R Egan[1], O Luton[2] , L Hopkins[2], O James[2], D Robinson[2], C Brown[2], S Hemington-Gorse[1], L Walsh[3], D Bailey[4], R Crane[5], C Lebares[6], W Lewis[2].

[1] Swansea Bay University Health Board, Morriston Hospital, Swansea, SA6 6NL, UK.
[2] Health Education and Improvement Wales' School of Surgery, Ty Dysgu, Cefn Coed, Nantgarw, CF15 7QQ, UK.
[3] Professional Support Unit, Health Education and Improvement Wales, Ty Dysgu, Cefn Coed, Nantgarw, CF15 7QQ, UK.
[4] Faculty of Life Sciences and Education, University of South Wales, Pontypridd, CF37 4AT, UK.
[5] Centre for Mindfulness Research and Practice, Canolfan Ymchwil ac Ymarfer Ymwybyddiaeth Ofalgar, Prifysgol Bangor University, Bangor, Gwynedd, LL57 2AS, UK
[6] University of California San Francisco Centre for Mindfulness in Surgery, 513 Parnassus Avenue, U-373, San Francisco, California, 94143, USA.

Background and Rationale

The current demands of surgical training produce work related pressure and emotional stress, which not only affect the well-being of trainees, but likely influence key assessment metrics, clinical performance, and ultimately threaten patient safety and quality of care.

Burnout among trainees is becoming increasingly recognised with symptoms akin to acute stress reaction (ASR) and post-traumatic stress disorder (PTSD) [1] and in the UK, some two thirds of junior doctors report damaged physical or mental health, because NHS work is associated with intense physical and emotional burdens and intolerable strain [2]. In Wales, 59% of surgical trainees reported burnout symptoms with Core Surgical Trainees (CST) reporting the highest risk [3]. Social relationships consequently suffer, and work related stress has even been reported to increase the risk of ischaemic heart disease, stroke [4-6], substance abuse, absenteeism, divorce, depression and suicide[7-9]. Moreover, such issues have been reported in as many as 70% of general surgery US residents[10-13] and 21% of mixed-specialty Dutch post-graduate trainees[14]. Occult trainee psychological morbidity therefore, may arguably be worse than that of the general population[1]. Shanafelt et al described almost 1 in 10 US surgeons (9%) self-reported major medical errors, and burnout and depression were shown to be independent predictors of error reporting, which correlated with all 3 burnout domains[15]. Similarly, emergency medicine physicians (EP), have been reported to have burnout rates of over 50%, and EPs with high burnout were significantly more likely to report clinical malpractice[16].

Among surgical trainees, high dispositional mindfulness decreases these risks by 75% or more [17], and formal mindfulness training has been shown feasible and acceptable [18-19]. In other high-stress populations such as the US Marine Corps, Police Force, and elite athletes [20-23], formal mindfulness training has improved well-being, stress, cognition and performance, yet the ability of such training to mitigate stress and burnout across medical specialties, or to affect improvements in the cognition and performance of physicians,

remains unknown. To address these gaps and thereby promote the wider adoption of contemplative practices within medical training, investigators have developed Enhanced Stress Resilience Training (ESRT), a modified form of Mindfulness-Based Stress Resilience (MBSR) - streamlined, tailored and contextualised for clinicians.

To date, no UK trainee and curriculum focused initiative exists, and although awareness is important, future work should focus on education and early identification to support trainees at risk of burnout.

Hypothesis
The primary hypothesis is that mindfulness-based Enhanced Stress Resilience Training (ESRT) is feasible for CSTs, can form part of a prescribed curriculum, and will reduce workplace associated stress and burnout.

A secondary hypothesis is that cognitive skills developed by ESRT will result in beneficial effects apparent in multiple domains including, psychosocial well-being, cognition, neural plasticity, and CST clinical performance metrics, as measured by professional examination success, ARCP Outcome, and promotion to higher surgical specialty training.

Study type
Prospective observational cohort study

Study population
Core surgical trainees (CST)

Methodology
Intervention:
Forty volunteers to be recruited from current cohort of CST. The intervention will be the modified form of Mindfulness-Based Stress Reduction (MBSR) named Enhanced Stress Resilience Training (ESRT). ESRT involves a 'learning component' consisting of five 60-minute group classes and one, two to three hour retreat (this will have to postponed until after COVID-19 social distancing measures are revoked). Classes focus on developing mindfulness skills (i.e. sustained attention and open monitoring) in addition to training focused on stress and coping. ESRT also involves a 'practice component': 3 to 20 minutes per day of mindfulness exercises following guided meditation media. The central exercises of ESRT are the body scan, sitting meditation and limited yoga. The weekly teaching sessions will take place on a workday evening. Daily practice will occur independently, unsupervised, with the duration reported daily.
Evaluation:
Cognitive and psychological testing will occur on three different occasions: pre-intervention, post-intervention and at six-month follow-up. The following validated psychosocial wellbeing surveys will be used, based around sensitivity demonstrated in prior work [18,19].

- Cognitive and Affective Mindfulness Scale – Revised (CAMS-R)
- Perceived Stress Scale (PSS-10)
- abbreviated Maslach Burnout Inventory (validated 2-item)
- Patient Health Questionnaire (PHQ-9)

- Speilberger State-Trait Anxiety Scale – SF (STAI)
- Mental Health Continuum Scale
- Alcohol Use Disorders Identification Test (AUDIT)
- Swedish Demand-Control-Support Scale

Cognitive testing will be undertaken using A computer and paper-based battery comprised of tasks targeting six cognitive domains (working memory, inhibition, set shifting, fluency, insight, and planning).
Performance of trainees will be assessed using Annual Review of Competence Progression (ARCP) outcomes and intercollegiate examination performance. ARCP outcomes will be based on a summation of several performance metrics, including logbook numbers, work based assessments completed, and consultant feedback (Multi-Consultant Report).
The Professional Support Unit at Health Education and Improvement Wales (HEIW) has granted full support for the study.

Statistical analysis
All outcomes (from questionnaires, tests and assays) will be summarised at each measurement time point. Statistical analysis appropriate for non-parametric data will be performed. Qualitive methods will be used to assess the feasibility of the intervention using thematic analysis. Primary satisfaction will be assessed using ordinal scales (Likert) and non-parametric tests.

Consent
Participation in the study is voluntary and written consent will be obtained from all participants.
All data will be anonymised and stored on password protected computers kept in a locked NHS office.

Expected study outcomes
The aim of the intervention is to provide CSTs with non-operative training skills that will serve them well and develop independently throughout their careers, with commensurate benefits to their health and well-being. Potentially, the intervention, may lead to enhanced focus and attention and even improved performance. By taking part in a study with the aim of benefiting all postgraduate medical trainees, participants should gain satisfaction from contributing to acquisition of knowledge in this field.

It is anticipated that the results of this study will be presented at international meetings of learned societies and published in peer reviewed journals.

Costs
Salary: Three fellows funded by Swansea Bay UHB, Cwm Taf Morgannwg UHB, Betsi Cadwaladr UHB for 2 years at mid-range of HST scale (ST5). One fellow jointly funded by Royal College of Surgeons of England/ Health Education and Improvement Wales for 1 year at mid-range of HST scale. Total funding secured £432K.
Delivery of ESRT: Two mindfulness coaches to deliver ESRT programme as described, including the necessary preparation time, cost £2000.

Laboratory costs: Hair cortisol analysis, total of 120 samples at a cost of £3600. Blood analysis, C-Reactive Protein (CRP) £120, Epigenetic Profile analysis £1800.

1. Thompson, C., Naumann, D., Fellows, J., Bowley, D. and Suggett, N. (2017). Post-traumatic stress disorder amongst surgical trainees: An unrecognised risk? The Surgeon, 15(3), pp.123-130.
2. Campbell, D. (2017). Two-thirds of young hospital doctors under serious stress, survey reveals. The Guardian. [online] Available at: http://www.theguardian.com/uk-news/2017/feb/11/stressed-out-docto rs-anaesthetists-survey-nhs [Accessed 31 May 2017].
3. Robinson DBT, James OP, Hopkins L, Brown C, Bowman C, Abdelrahman T, Pollitt MJ, Egan RJ, Bailey DM, Lewis WG. Stress and Burnout in Training; Requiem for the Surgical Dream. Journal of Surgical Education. 2019 https://doi.org/10.1016/j.jsurg.2019.07.002
4. Eller, N., Netterstrøm, B., Gyntelberg, F., Kristensen, T., Nielsen, F., Steptoe, A. and Theorell, T. (2009). Work-Related Psychosocial Factors and the Development of Ischemic Heart Disease. Cardiology in Review, 17(2), pp.83-97.
5. Kivimäki, M., Virtanen, M., Elovainio, M., Kouvonen, A., Väänänen, A. and Vahtera, J. (2006). Work stress in the etiology of coronary heart disease—a meta-analysis. Scandinavian Journal of Work, Environment & Health, 32(6), pp.431-442.
6. Booth, J., Connelly, L., Lawrence, M., Chalmers, C., Joice, S., Becker, C. and Dougall, N. (2015). Evidence of perceived psychosocial stress as a risk factor for stroke in adults: a meta-analysis. BMC Neurology, 15(1).
7. Dyrbye LN, Shanafelt TD, Balch CM, et al. Relationship be- tween work-home conflicts and burnout among American surgeons: a comparison by sex. Arch Surg 2011;146:211e217.
8. Oreskovich MR, Kaups KL, Balch CM, et al. Prevalence of alcohol use disorders among American surgeons. Arch Surg 2012;147:168e174.
9. Shanafelt TD, Balch CM,Dyrbye L et al. Special report: suicidal ideation among American surgeons. Arch Surg 2011;146:54e62.
10. Elmore, L., Jeffe, D., Jin, L., Awad, M. and Turnbull, I. (2016). National Survey of Burnout among US General Surgery Residents. Journal of the American College of Surgeons, 223(3), pp.440-451.
11. Clever LH. Who is sicker: patients--or residents? Residents' distress and the care of patients. Ann Intern Med 2002;136:391–393.
12. Girard DE, Hickam DH, Gordon GH, Robison RO. A prospective study of internal medicine residents' emotions and attitudes throughout their training. Acad Med 1991;66:111–114.
13. West CP, Shanafelt TD, Kolars JC. Quality of life, burnout, educational debt, and medical knowledge among internal medicine residents. JAMA 2011;306:952–960
14. Prins, J., Hoekstra-Weebers, J., Gazendam-Donofrio, S., Dillingh, G., Bakker, A., Huisman, M., Jacobs, B. and van der Heijden, F. (2010). Burnout and engagement among resident doctors in the Netherlands: a national study. Medical Education, 44(3), pp.236-247.
15. Shanafelt, T., Balch, C., Bechamps, G., Russell, T., Dyrbye, L., Satele, D., Collicott, P., Novotny, P., Sloan, J. and Freischlag, J. (2010). Burnout and Medical Errors Among American Surgeons. Annals of Surgery, 251(6), pp.995-1000.
16. Lu, D., Dresden, S., McCloskey, C., Branzetti, J. and Gisondi, M. (2015). Impact of Burnout on Self-Reported Patient Care Among Emergency Physicians. Western Journal of Emergency Medicine, 16(7), pp.996-1001.

17. Lebares CC, Guvva EV, Ascher NL, O'Sullivan PS, Harris HW, Epel ES. Burnout and Stress Among US Surgery Residents: Psychological Distress and Resilience. J Am Coll Surg. 2018;226(1): 80-90.
18. Lebares CC, Guvva EV, Olaru M, Sugrue LP, Staffaroni AM, Delucchi KL, Kramer JH, Ascher NL, Harris HW. Efficacy of Mindfulness-Based Cognitive Training in Surgery: Additional Analysis of the Mindful Surgeon Pilot Randomized Clinical Trial. JAMA Netw Open. 2019 May 03; 2(5):e194108.
19. Lebares CC, Hershberger AO, Guvva EV, Desai A, Mitchell J, Shen W, Reilly LM, Delucchi KL, O'Sullivan PS, Ascher NL, Harris HW. Feasibility of Formal Mindfulness-Based Stress-Resilience Training Among Surgery Interns: A Randomized Clinical Trial. JAMA Surg. 2018 10 01; 153(10):e182734.
20. Johnson DC, Thom NJ, Stanley EA, et al. Modifying resilience mechanisms in at-risk individuals: a controlled study of mindfulness training in Marines preparing for deployment. *Am J Psychiatry.* 2014;171(8):844-853.

21. Le Scanff C, Taugis J. Stress management for police special forces. *J App Sport Psych.* 2002;14(4):330-343.

22. Guenthner SV, Hammermeister J, Burton D, Keller L. Smoke and mirrors or wave of the future? Evaluating a mental skills training program for elite cross- country skiers. *J Sport Behav.* 2010;33(1):3-24.

23. Birrer D, Röthlin P, Morgan G. Mindfulness to enhance athletic performance: Theoretical considerations and possible impact mechanisms. *Mindfulness*. 2012;3:235–246.
